# Supplementary material for: Home-Based Stair Climbing as an Intervention for Disease Risk in Adult Females; A Controlled Study
Source: Int J Environ Res Public Health. 2021 Jan 12;18(2):603. doi: 10.3390/ijerph18020603 (PMC7828146; doi:10.3390/ijerph18020603)
Supplement: Supplementary file 1 [file ijerph-18-00603-s001.pdf]

Supplementary Table S1: Medians (IQR) pre and post for the control, gym-based and home-based groups, with a summary of non-parametric statistical testing.

Variable	Control (n=10)		Gym-based (n=24)		Home-based (n=26)		Change Pre:post x Cnt:Exp[a]	Stair group Pre:post	Location x change Pre:post
	Pre (IQR)	Post (IQR)	Pre (IQR)	Post (IQR)	Pre (IQR)	Post (IQR)	Fligner-Wolfe standardized	Wilcoxon test Z statistic	Kruskal-Wallis test value
$\dot{V}O_2max$ ($ml.min^{-1}.kg^{-1}$)	24.20 (1.70)	24.65 (2.60)	25.00 (5.65)	26.10 (6.30)	23.80 (4.00)	24.70 (4.20)	**2.37**[bc]**	**5.39*** **	0.13
Rating of perceived exertion	17.00 (5.00)	17.50 (4.00)	17.00 (7.00)	17.00 (6.00)	19.00 (5.00)	18.00 (3.00)	0.38	0.65	0.10
Triglycerides ($mmol.L^{-1}$)	0.93 (0.36)	0.75 (0.63)	1.17 (1.25)	1.06 (1.01)	1.49 (0.92)	1.29 (0.66)	**2.46****	**5.37*** **	2.24
Weight (kg)	52.45 (4.90)	52.50 (4.50)	71.55 (25.33)	70.75 (26.13)	80.30 (31.30)	78.80 (31.70)	**2.58***	**4.42*** **	0.73
Body fat (%)	22.03 (5.85)	21.88 (3.27)	28.55 (7.79)	27.35 (10.08)	32.24 (7.70)	27.90 (9.99)	**2.00***	**5.42*** **	0.19

a: cnt = control; exp = experimental. b: † $p \leq .10$, * $p \leq .05$, ** $p \leq .01$, *** $p \leq .001$. c: Significant effects with two-tailed probabilities are presented in bold.

For the test of improvement relative to controls, the changes pre-post were compared with the Fligner-Wolfe test (Hollander & Wolfe, 1999). It was of interest that *improvements relative control* for body fat were found, unlike the parametric analysis. The Wilcoxon test compared pre with post values in the stair group alone. All of these changes to outcome variables were significant at $p < .001$. For any differences by location, the Mann-Whitney test of the changes pre vs. post, with the Kruskal Wallis test statistic reported.

Hollander, M. & Wolfe, D.A. (1999). *Non-parametric statistical methods*, 2nd ed. New York, John Wiley & Sons.
